# Two Ensemble-CNN Approaches for Colorectal Cancer Tissue Type Classification

**DOI:** 10.3390/jimaging7030051

**Published:** 2021-03-09

**Authors:** Emanuela Paladini, Edoardo Vantaggiato, Fares Bougourzi, Cosimo Distante, Abdenour Hadid, Abdelmalik Taleb-Ahmed

**Affiliations:** 1 Department of Innovation Engineering, University of Salento, 73100 Lecce, Italy; emanuela.paladini@studenti.unisalento.it (E.P.); edoardo.vantaggiato@studenti.unisalento.it (E.V.); 2 Univ. Polytechnique Hauts-de-France, Univ. Lille, CNRS, Centrale Lille, UMR 8520—IEMN, F-59313 Valenciennes, France; faresbougourzi@gmail.com (F.B.); abdenour.hadid@ieee.org (A.H.); 3 Institute of Applied Sciences and Intelligent Systems, National Research Council of Italy, 73100 Lecce, Italy

**Keywords:** digital pathology, colorectal cancer, tissue phenotyping, convolutional neural network, ensemble CNN

## Abstract

In recent years, automatic tissue phenotyping has attracted increasing interest in the Digital Pathology (DP) field. For Colorectal Cancer (CRC), tissue phenotyping can diagnose the cancer and differentiate between different cancer grades. The development of Whole Slide Images (WSIs) has provided the required data for creating automatic tissue phenotyping systems. In this paper, we study different hand-crafted feature-based and deep learning methods using two popular multi-classes CRC-tissue-type databases: Kather-CRC-2016 and CRC-TP. For the hand-crafted features, we use two texture descriptors (LPQ and BSIF) and their combination. In addition, two classifiers are used (SVM and NN) to classify the texture features into distinct CRC tissue types. For the deep learning methods, we evaluate four Convolutional Neural Network (CNN) architectures (ResNet-101, ResNeXt-50, Inception-v3, and DenseNet-161). Moreover, we propose two Ensemble CNN approaches: Mean-Ensemble-CNN and NN-Ensemble-CNN. The experimental results show that the proposed approaches outperformed the hand-crafted feature-based methods, CNN architectures and the state-of-the-art methods in both databases.

## 1. Introduction

Traditionally, pathologists have used the microscope to analyze the micro-anatomy of cells and tissues. In recent years, the advancement in Digital Pathology (DP) imaging has provided an alternative way to enable the pathologists to do the same analysis over the computer screen [1]. The new DP imaging modality is able to digitize the Whole Slide Imaging (WSI), where the glass slides are converted into digital slides that can be viewed, managed, shared and analyzed on a computer monitor [2].

In Colorectal Cancer (CRC), tumor architecture changes during tumor progression [3] and is related to patient prognosis [4]. Therefore, quantifying the tissue composition in CRC is a relevant task in histopathology. Tumor heterogeneity occurs both between tumors (inter-tumor heterogeneity) and within tumors (intra-tumor heterogeneity). In fact, Tumor Micro-Environment (TME) plays a crucial role in the development of Intra-Tumor Heterogeneity (ITH) by the various signals that cells receive from their micro-environment [5].

Colorectal Cancer (CRC) is considered as the fourth most occurring cancer and it is the third leading cancer type to cause death [6]. Indeed, early stage CRC diagnosis is decisive for therapy of patients and saving their lives [7]. The evaluation of tumor heterogeneity is very important for cancer grading and prognostication [8]. In more detail, intre-tumor heterogeneity can aid the understanding of TME’s effect on patient prognosis, as well as identify novel aggressive phenotypes that can be further investigated as potential targets for new treatment [9].

In recent years, automatic tissue phenotyping, in Whole Slide Images (WSIs), has become a fast-growing research area in computer vision and machine learning communities. In fact, state-of-the-art approaches have investigated the classification of two tissue types [10,11] or multi-class tissue types analysis [8,12,13]. The two tissue types are tumor and stroma tissue categories. Actually, the classification of just two tissue categories is not suitable for more heterogeneous parts of the tumor [12]. To overcome this limitation, the authors of [12] proposed the first multi-class tissue type database, where they considered eight tissue types.

In this work, we deal with the classification of multi-class tissue types. In order to classify different CRC tissue types, we proposed two ensemble approaches which are: Mean-Ensemble-CNNs and NN-Ensemble-CNNs. Our proposed approaches are based on combining four trained CNN architectures, which are ResNet-101, ResNeXt-50, Inception-v3 and DenseNet-161. Our Mean-Ensemble-CNN approach uses the predicted probabilities of different trained CNN models. On the other hand, the NN-Ensemble-CNN approach used combined deep features that were extracted from different trained CNN models, then classified them using NN architecture. Since automatic multi-class CRC tissue classification is a relatively new task, we evaluated two hand-crafted descriptors which are: LPQ and BSIF. In addition, two classifiers were used which are SVM and NN. As summary, the main contributions of this paper are:

- We proposed two ensemble CNN-based approaches: Mean-Ensemble-CNNs and NN-Ensemble-CNNs. Both of our approaches combine four trained CNN architectures which are ResNet-101, ResNeXt-50, Inception-v3 and DenseNet-161. The first approach (Mean-Ensemble-CNNs) uses the predicted probabilities of the four trained CNN models to classify the CRC tissue types. The second approach (NN-Ensemble-CNNs) combines the deep features that were extracted using the trained CNN models, then it uses NN architecture to recognize the CRC phenotype.
- We conducted extensive experiments to study the effectiveness of our proposed approaches. To this end, we evaluated two texture descriptors (BSIF and LPQ) and their combination using two classifiers (SVM and NN) in two CRC tissue types databases.
- Implicitly, our work contains comparison between CNN architectures and hand-crafted feature-based methods for the classification of CRC tissue types using two publicly databases.

This paper is organized as follows: In Section 2, we describe the state-of-the-art methods. Section 3 includes description of the used databases, methods and evaluation metrics. In addition, Section 3 contains an illustration of our proposed approach and experimental setup. Section 4 represents the experimental results. In Section 5, we compare our results with the state-of-the-art methods. Finally, we conclude our work in Section 6.

## 2. Related Works

In recent years, CRC tissue phenotyping has been subject to increasing interest in both computer vision and machine learning fields due to the availability of CRC-tissue-type databases such as [8,12,14,15]. Supervised methods are widely used to classify the tissue types in histological images [12]. The supervized state-of-the-art methods for phenotyping the CRC tissues can be categorized as texture [10,11,12,16], or learned methods [8,15,17,18]. In addition, there are some works that combined deep and shallow features such as [19]. The texture methods are hand-crafted algorithms that were designed based on mathematical model to extract specific structures within the image regions [20]. However, deep learning methods have the ability to learn more relevant and complex features directly from the images across their layers. In particular, when there is no prior knowledge about the relationship between input data and the outcomes to be predicted. Since the pathology imaging tasks are very complex and little is known about which quantitative image features predict the outcomes, deep learning methods are suitable for these tasks [21,22]. In this section, we will describe the state-of-the-art works that have addressed multi-class CRC tissue types and used supervized methods.

In [12], J. N. Kather et al. were the first who addressed CRC multi-class tissue types, where they created their database from 5000 histological images of human colorectal cancer including eight CRC tissue types. J. N. Kather et al. tested several state-of-the-art texture descriptors and classifiers. Their proposed approach is based on the combination GLCM and LBP local descriptors beside with global lower-order texture measures which achieved promising performance. In [19], Nanni et al. proposed the General Purpose (GenP) approach which is based on ensemble of multiple hand-crafted, dense sampling and learned features. In their combined approach, they trained each feature using SVM then combined all of them using the sum rule. Cascianelli et al. [23] compared deep and shallow features to recognize the CRC tissue types. In their work, they studied the impact of using dimensionality reduction strategies in both accuracy and computational cost. Their results showed that the best trade-off between accuracy and dimensionality using CNN-based features is possible.

In [15], J. N. Kather et al. used 86 H&E slides of CRC tissues from the NCT biobank and the UMM pathology to create a training image set of 100,000 images that were labeled into eight tissue types. They tested five pretrained CNN models: VGG19 [24], AlexNet [25], SqueezeNet version 1.1 [26], GoogLeNet [27], and ResNet-50 [28]. They concluded that VGG19 was the best model among the five CNN models. Javed et al. [8] proposed a new CRC-TP database which consists of 280K patches extracted from 20 WSIs of CRC; these patches are classified into seven distinct tissue phenotypes. To classify these tissue types, they used 27 state-of-the-art methods including texture, CNN and Graph CNN-based (GCN) methods. From their experimental results, the GCN outperformed the texture and CNN methods. Despite hand-crafted feature-based and deep learning methods having been used for multi-class CRC tissue type classification, the performance of these methods still needs more improvement. To this end, we proposed two ensemble-CNN approaches that achieved considerable improvement on two popular databases.

## 3. Methodology

### 3.1. Databases

#### 3.1.1. Kather-CRC-2016 Database

Kather-CRC-2016 database [12] consists of 5000 CRC tissue type images, where each tissue type has 625 samples. J. N. Kather et al. [12] used 10 anonymized H&E stained CRC tissue slides from the pathology archive at the University Medical Center Mannheim, Germany. Firstly, they digitized the slides. Then, contiguous tissue areas were manually annotated and tessellated. From each slide, they created 625 non-overlapping tissue tiles of dimension 150×150 pixels. The following eight types of tissues were selected in their database:

(a) Tumor epithelium.
(b) Simple stroma (homogeneous composition, includes tumor stroma, extra-tumoral stroma and smooth muscle).
(c) Complex stroma (containing single tumor cells and/or few immune cells).
(d) Immune cells (including immune-cell conglomerates and sub-mucosal lymphoid follicles).
(e) Debris (including necrosis, hemorrhage and mucus).
(f) Normal mucosal glands.
(g) Adipose tissue.
(h) Background (no tissue).

**Figure 1:**
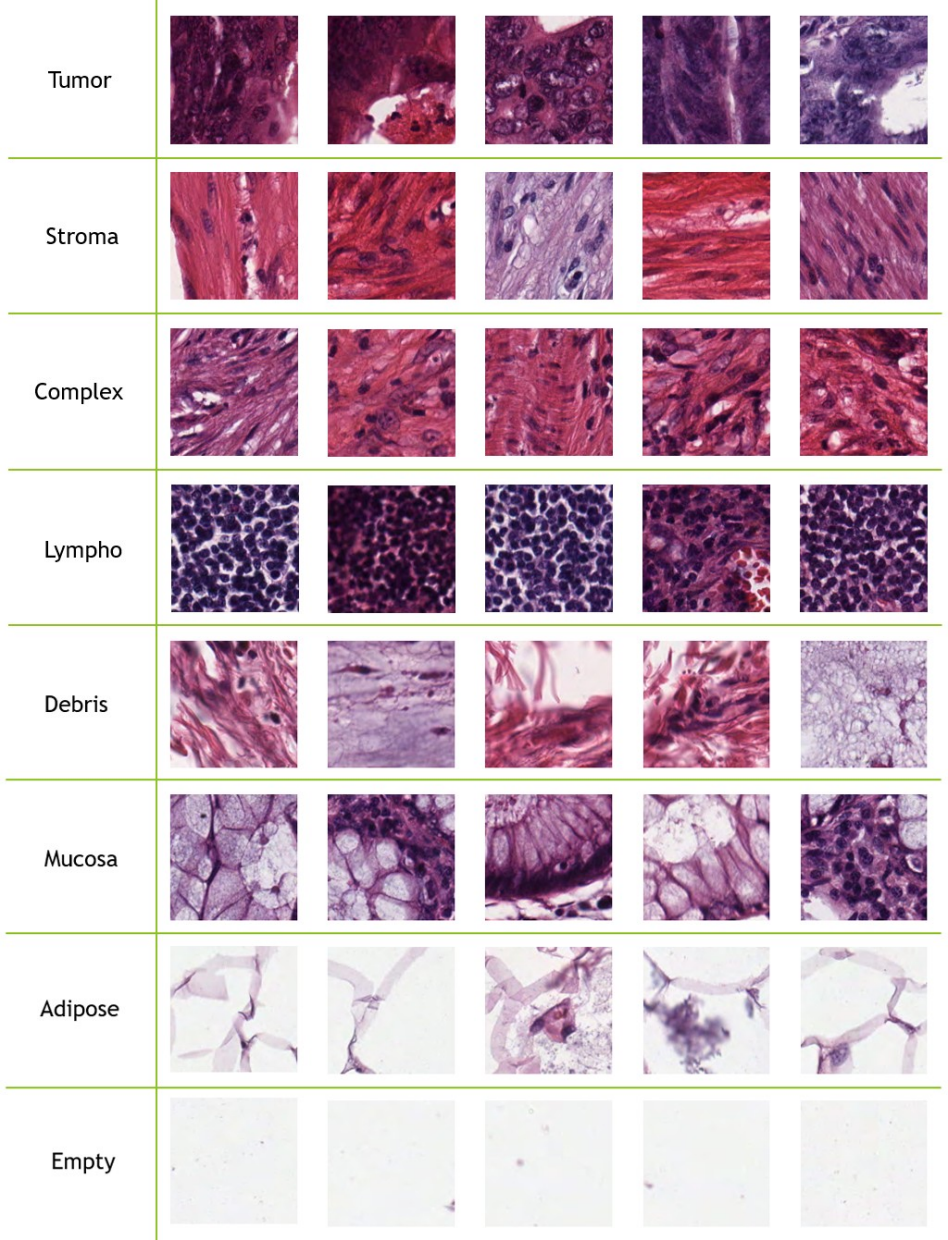
Five samples for each class from the Kather-CRC-2016 database [12].

Figure 1 contains five samples for each CRC tissue type from the Kather-CRC-2016 database. J. N. Kather et al. [12] used 10-fold cross validation to evaluate texture methods.

#### 3.1.2. CRC-TP Database

The CRC-TP database [8] consists of 280K CRC tissue type images. These CRC tissue type images are patches that were extracted from 20 WSIs of CRC stained with H&E taken from University Hospitals Coventry and Warwickshire (UHCW). Each slide was taken from a different patient. With the aid of expert pathologists, the WSI slides were manually divided into non-overlapping patches and these patches were annotated into seven distinct tissue phenotypes, where each patch was assigned to a unique label based on the majority of its content. Table 1 contains the CRC tissue types and their corresponding number of samples.

**Figure 2:**
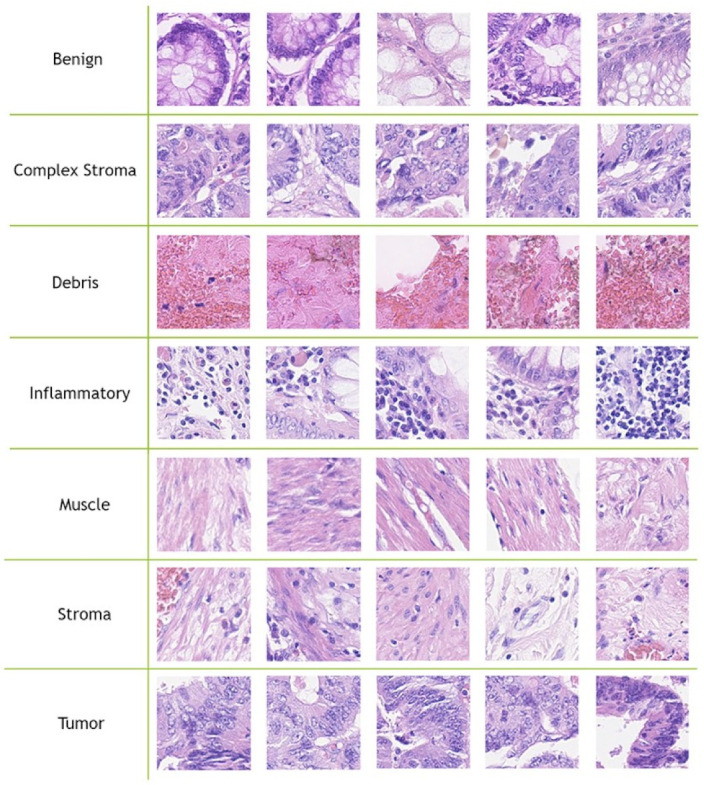
Five samples for each class from the CRC-TP database [8].

The CRC tissue image size is fixed to 150×150 pixels. Javed et al. [8] divided the 280K CRC tissue images into training and testing splits to evaluate the performance of their methods, where 70% of each tissue phenotype patches are randomly selected as the training split and the remaining 30% are used as testing split. In our experiments, we used the provided patch-level separation data splits (70–30%) that were provided by [8]. Figure 2 contains five samples of each CRC tissue type from the CRC-TP database.

### 3.2. Hand-Crafted Methods

#### 3.2.1. Local Phase Quantization (LPQ)

In the last three decades, texture descriptors have proved their efficiency in many computer vision tasks. In our experiments, we used two of the most powerful descriptors: Local Phase Quantization (LPQ) [29] and Binarized Statistical Image Features (BSIF) [30]. In addition, we tested the combination of these two descriptors by concatenating their features alongside each other.

**Figure 3:**
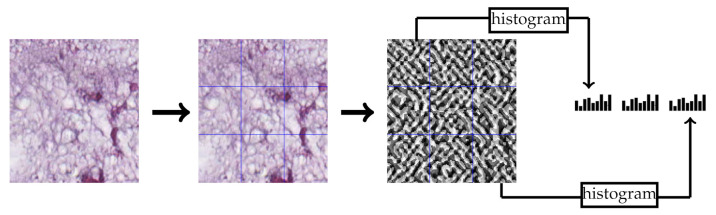
Multi-block LPQ feature extraction example of 3×3 multi-block representation.

LPQ [29] is a local texture descriptor based on quantized phase of the Discrete Fourier Transform (DFT) in local neighborhood pixels. For local neighborhood pixels M×M, short-term Fourier transform is used to quantize the phase of Fourier transform by considering four frequencies. In our experiments, we choose LPQ parameters as follows: the local neighborhood size of the block is 13×13 pixels, the frequency estimation method is the Gaussian derivative quadrature filter pair and 3×3 multi-block representation. Each block produces a histogram which contains the repetition of the quantized phases for all pixels within this block. Consequently, each block produces a 256-dimensional feature vector and the final feature vector is the concatenation of all block feature vectors. Figure 3 contains an example of extracting the LPQ features from a CRC tissue image.

#### 3.2.2. Binarized Statistical Image Features (BSIF)

**Figure 4:**
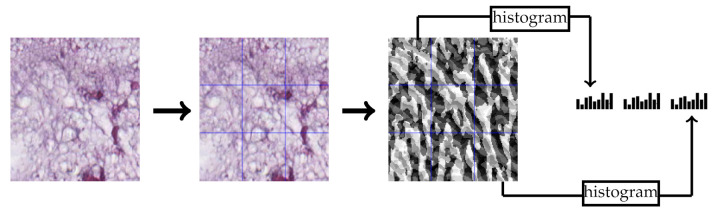
Multi-block BSIF feature extraction example of 3×3 multi-block representation.

BSIF [31] is a local texture descriptor that uses a set of 2-D filters to have a binarized response for each pixel. These filters were learned from natural images using independent component analysis. In our experiments, we used the 17×17×8 bank of filters. Similar to LPQ feature extraction, we used the 3×3 multi-block representation. Each block produces a 256-dimensional feature vector and the final feature vector is the concatenation of all block feature vectors. Figure 4 contains an example of extracting the BSIF features from a CRC tissue image.

#### 3.2.3. Support Vector Machine (SVM)

**Figure 5:**
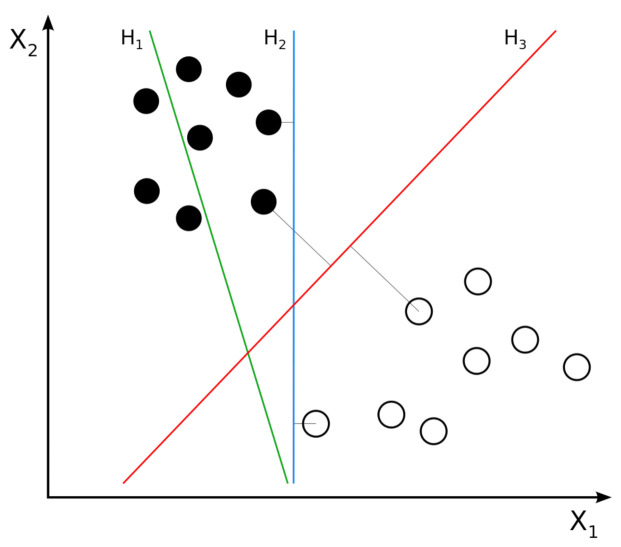
Example of two classes of linear SVM. H1 does not separate the classes. H2 separates the classes, but only with a small margin. H3 separates the classes with the maximal margin.

In machine learning, SVM [32] is one of the most powerful supervized learning methods. For D features, the SVM algorithm seeks to define a hyperplane in D-dimensional space that distinctly classifies the data points. To separate two classes of data points, there are many possible hyperplanes that could be chosen. SVM objective is to find the plane that has the maximum margin, i.e., the maximum distance between data points of all classes. Maximizing the margin distance provides some reinforcement so that future data points can be classified with more confidence. Figure 5 shows an example of possible hyperplanes between two classes and the linear-SVM hyperplane, which separates the two classes of data points with maximum margin. In our experiments, we used linear-SVM as a benchmark classifier for the hand-crafted features.

#### 3.2.4. Neural Network (NN) Classifier

**Figure 6:**
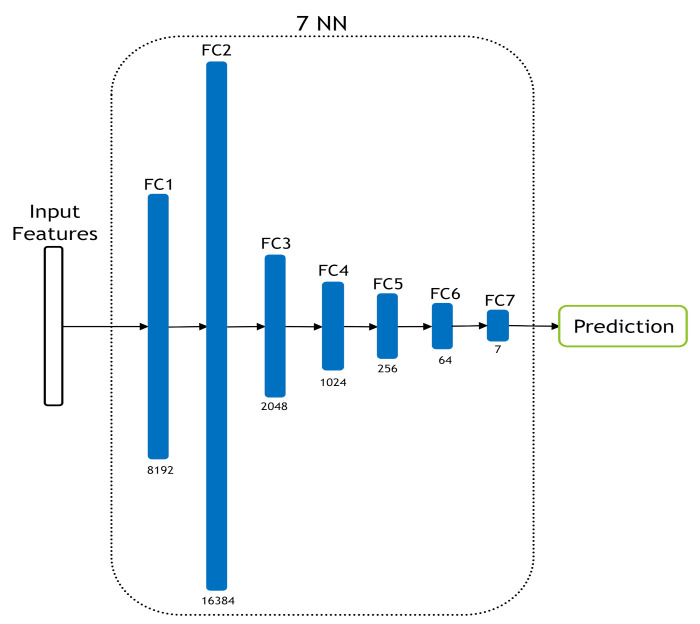
The used seven-layer NN classifier.

In addition to the SVM classifier, we built a seven-layer NN classifier to classify the shallow features that were obtained from LPQ and BSIF descriptors and their combination. Figure 6 illustrates the used architecture. We chose these seven layers to make the classifier simple since the extracted features are already middle-level features. To this end, we tested a different number of layers (3, 5, 7 and 9) on the first fold of the Kather-CRC-2016 database, then we picked out the number of layers corresponding to the best performance which was seven layers. Consequently, the seven-layer NN architecture was used in the other folder of the Kather-CRC-2016 database and CRC-TP database experiments. The seven-layer NN classifier was trained for 20 epochs, initial $lr=10^{-6}$ with decay of 0.1 every 10 epochs and batch size equals 128.

### 3.3. CNN Architectures

In our experiments, we evaluated four of the most powerful CNN architectures, which are: ResNet-101, ResNeXt-50, Inception-v3, and DenseNet-161. Here, we used the pre-trained models that were trained on ImageNet challenge database [25].

#### 3.3.1. ResNet-101

**Figure 7:**
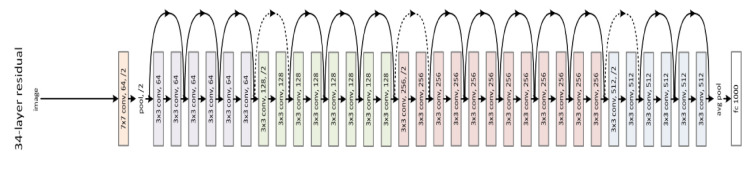
Example of 34 residual layers.

The traditional CNN architectures suffer from gradient vanishing/exploding when going deeper. In [28], K. He et al. proposed a solution to the gradient vanishing/exploding problem by using residual connections straight to earlier layers as shown in Figure 7. The residual networks are easier to optimize, and can gain accuracy from considerably increased depth with lower complexity than the traditional CNNs. In our experiments, we used the ResNet-101 pretrained model.

#### 3.3.2. ResNeXt-50

**Figure 8:**
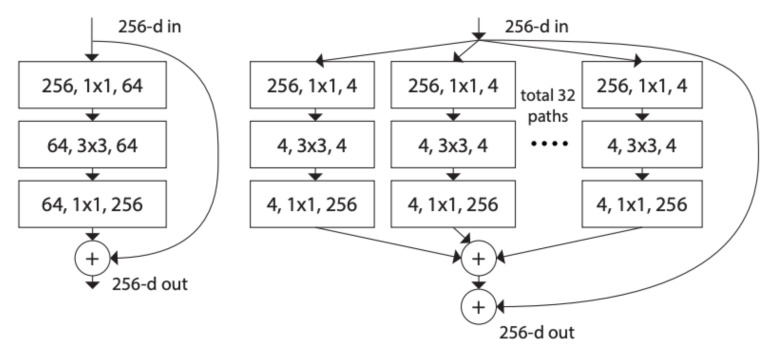
ResNet and ResNeXt blocks [33].

ResNeXt block [33] uses the residual connections straight to earlier layers similar to ResNet block as shown in Figure 8. In addition, ResNeXt block adopts the split–transform–merge strategy (branched paths within a single module). In the ResNeXt block, as shown in Figure 8, the input is split into many lower-dimensional embeddings (by 1 × 1 convolutions)—32 paths each of 4 channels; then all paths are transformed by the same topology filters of size 3×3. Finally, the paths are merged by summation.

#### 3.3.3. Inception-v3

**Figure 9:**
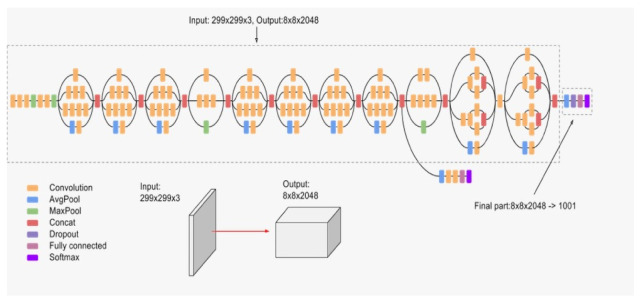
Inception architecture.

Inception-v3 [34] is the third version of the Inception networks family that were introduced first hand in [27]. Inception block provides efficient computation and deeper networks through a dimensionality reduction with stacked 1×1 convolutions. The main idea of Inception architectures is to make multiple kernel filter sizes operate on the same level instead of stacking them sequentially as was the case in the traditional CNNs. This is known as making the networks wider instead of deeper. Figure 9 illustrates the architecture of Inception-v3, which makes several improvements compared to the initial Inception versions. These improvements include using label smoothing, factorized 7×7 convolutions, and the use of an auxiliary classifier to propagate label information lower down in the network.

#### 3.3.4. DenseNet-161

**Figure 10:**
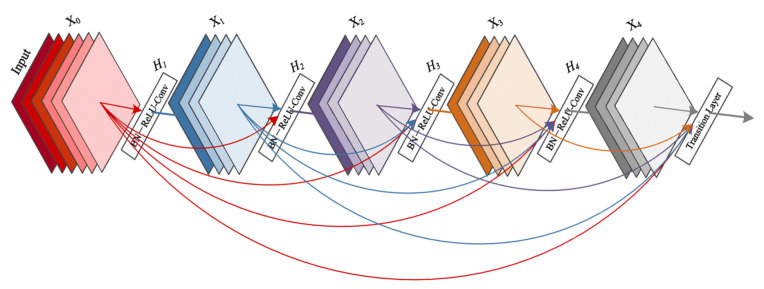
A 5-layer dense block with a growth rate of k = 4. Each layer takes all preceding feature maps as input [35].

DenseNet networks [35] seek to solve the problem of CNNs when going deeper. This is because the path for information from the input layer until the output layer (and for the gradient in the opposite direction) becomes so big, that it can vanish before reaching the other side. G. Huang et al. [35] proposed connecting each layer to every other layer in a feed-forward fashion (as shown in Figure 10) to ensure maximum information flow between layers in the network. In our experiments, we used the DenseNet-161 pre-trained model.

### 3.4. Focal Loss

**Figure 11:**
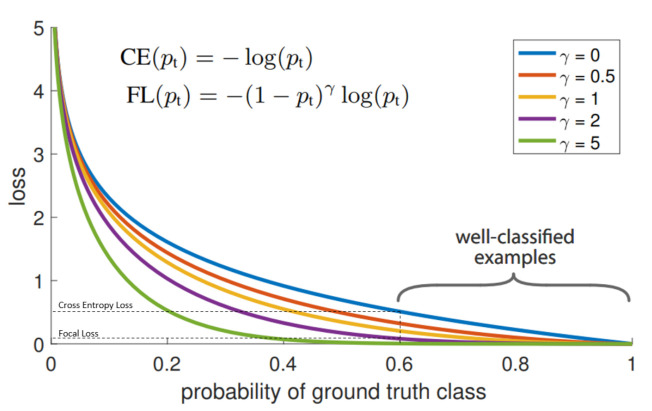
Comparison between Focal Loss with different focusing parameter γ values and the Cross-Entropy Loss function [36].

Originally, Focal Loss function was proposed for one-stage object detectors [36], where it proved its efficiency in the imbalanced classes case. The Focal Loss function is defined by:

(1) $$FL\left(p_{t}\right)=-{\left(1-p_{t}\right)}^{\gamma }log\left(p_{t}\right)$$

where: $p_{t}$ is the predicted probability corresponding to the ground truth class, γ is the focusing parameter. Figure 11 shows a comparison between the Cross-Entropy loss function and Focal Loss function with different values of focusing parameter γ. As shown in Figure 11, γ controls the shape of the curve. The higher the value of γ, the lower loss will be assigned to the well-classified examples. At γ=0, Focal Loss becomes equivalent to Cross Entropy Loss. In addition to one-stage object detection task, Focal loss function has proved its efficiency in many classification tasks [37,38].

### 3.5. Evaluation Metrics

To evaluate the performance of the tested methods, we used three metrics which are: accuracy, F1-score and $\hat{F1}$-score. Accuracy is the measurement of all correctly classified samples over the total number of samples. The accuracy is mainly used to evaluate the methods on the Kather-CRC-2016 database because it is a balanced database. Since the CRC-TP database is not a balanced database, we used F1-score and $\hat{F1}$-score. F1-score is defined by the formula:

(2) $$F1-\mathrm{score}=2*\frac{Precision*Recall}{Precision+Recall}$$

where: *Precision* and *Sensitivity* (also called *Recall*) are defined by the following formulas:

(3) $$Precision=\frac{TP}{TP+FP}$$

(4) $$Sensitivity=\frac{TP}{TP+FN}$$

where *TP* is the number of True Positive instances, *FP* is the number of False Positive instances and *FN* is the number of the False Negative instances.

$\hat{F1}$-score is defined by the formula:

(5) $$\hat{F1}-\mathrm{score}=\frac{\sum_{i}^{C}n_{i}{F1-\mathrm{score}}_{i}}{N}$$

where *C* is the number of classes, $n_{i}$ is the number of test samples of *i*-th class, and *N* is the total number of test samples.

### 3.6. Proposed Approaches

To classify different CRC tissue types, we propose two Ensemble-CNN approaches: Mean-Ensemble-CNNs and NN-Ensemble-CNNs. The proposed approaches used the already trained CNN models (ResNet-101, ResNeXt-50, Inception-v3 and DenseNet-161) for CRC tissue type classification using the training data.

**Figure 12:**
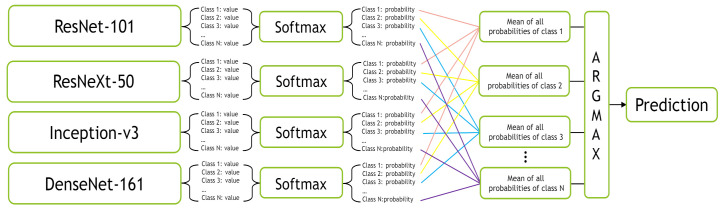
The proposed Mean-Ensemble-CNN approach.

In the Mean-Ensemble-CNN approach, the predicted class of each image is assigned using the average of the predicted probabilities of four trained models. In more detail, the probabilities of the four models corresponding to all classes give the mean probability for each class, then the max of the mean probabilities assigns the ensemble predicted class. Figure 12 illustrates our Mean-Ensemble-CNN approach.

**Figure 13:**
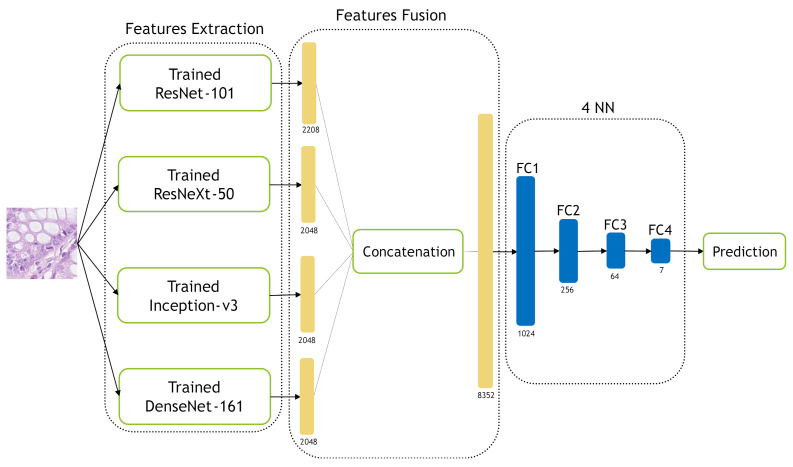
The proposed NN-Ensemble-CNN approach.

In the NN-Ensemble-CNN approach, the deep features corresponding to the last FC layer are extracted from the four trained models. Then, these deep feature vectors are concatenated alongside each other to obtain an ensemble deep feature vector. The extracted training features (from the training data) are used to train new NN architecture, which consists of four layers. On the other hand, the extracted testing features (from the testing data) are used to test the four-layer NNs. Figure 13 illustrates our NN-Ensemble-CNN approach. The selection of four layers for our NN-Ensemble-CNNs approach was after testing different small numbers of layers (3, 4 and 5) on the first fold of the Kather-CRC-2016 database. This was similar to what we did for the NN classifier of the hand-crafted features in Section 3.2.4.

### 3.7. Experimental Setup

For hand-crafted feature extraction and SVM classification, we used MATLAB 2019. For deep learning and NN training, we used the Pytorch [39] library with NVIDIA GPU Device Geforce TITAN RTX 24 GB. For training the deep learning architectures, we used data pre-processing including normalizing and resizing the input images to have the correct input size for each network. Inception-v3 input size is 299 × 299 pixels, while DenseNet-161, ResNeXt-50 and ResNet-101 need an input size of 224 × 224. Moreover, we used the following active data augmentation techniques:

- Random Cropping;
- Random Horizontal flip with applying probability = 0.2;
- Random Vertical flip with applying probability = 0.2;
- Random Rotation from −30 to 30 degree.

## 4. Experiments and Results

In this section, we will describe our experimental setup and the experimental results.

### 4.1. Hand-Crafted Feature Experiments

In this section, we used two hand-crafted descriptors (LPQ and BSIF) to extract the features from CRC tissue images. After the features were extracted, we used two classification methods (SVM and NN) to distinguish between different CRC phenotyping.

#### 4.1.1. Kather-CRC-2016 Database

In Kather-CRC-2016 database experiments, we used 5-fold cross-validation evaluation scheme. Table 2 summarizes the obtained accuracy for each fold and the mean of the five folds accuracies. From this table, we notice that the combination of LPQ and BSIF gave better performance for both classifiers (SVM and NN). On the other hand, we observe that combined features achieved similar results with the two classifiers, with slightly better accuracy with the seven-layer NN classifier.

#### 4.1.2. CRC-TP Database

In the CRC-TP database, we used the train and validation splits that were provided with the database [8]. Table 3 summarizes the obtained results of LPQ and BSIF descriptors and their combination using SVM and NN classifiers. Similar to Kather-CRC-2016 experiments, we noticed that the combination of LPQ and BSIF gave better performance for both classifiers (SVM and NN). In addition, we observed that NN classifier with the combined features achieved the best performance.

### 4.2. Deep Learning Experiments

In this section, we evaluated four CNN architectures which are ResNet-101, ResNeXt-50, Inception-v3, and DenseNet-161, and two proposed ensemble schemes which are Mean-Ensemble-CNNs and NN-Ensemble-CNNs. All Networks are trained for 20 epochs with an Adam optimizer [40] and Focal Loss function [36] with γ=2. The initial learning rate is $10^{-5}$ for 10 epochs, then the learning rate decreases to $10^{-6}$ for next 10 epochs. We also add a dropout layer in DenseNet-161, ResNeXt-50 and ResNet-101 before the fully connected layer with a probability of 0.3. Meanwhile, Inception-v3 already has a dropout layer with a probability of 0.5. For NN-Ensemble-CNNs, we used the four trained models to extract the deep features, then we trained the four-layer NN network as described in Figure 13. The four-layer NN network is trained for 30 epochs with an initial learning rate of $10^{-6}$ and it decays after 15 epochs. Similar to the CNN architectures, the four-layer NN network is trained using an Adam optimizer [40] and Focal Loss function [36] with γ=2.

#### 4.2.1. Kather-CRC-2016 Database

Similar to the hand-crafted feature experiments on the Kather-CRC-2016 database, we used a 5-fold cross-validation evaluation scheme. For training the CNN architectures, we used a batch size of 64. To train the NN network for our NN-Ensemble-CNN approach, we used a batch size of 32. Table 4 summarizes the obtained results using the CNN architectures and the proposed ensemble approaches. By comparing these results with the ones from Table 2, we notice that the CNN architectures exceed the hand-crafted feature-based methods in the classification of CRC tissue types. We noticed that the performance of the two proposed ensemble approaches outperformed the performance of the four CNN architectures.

**Figure 14:**
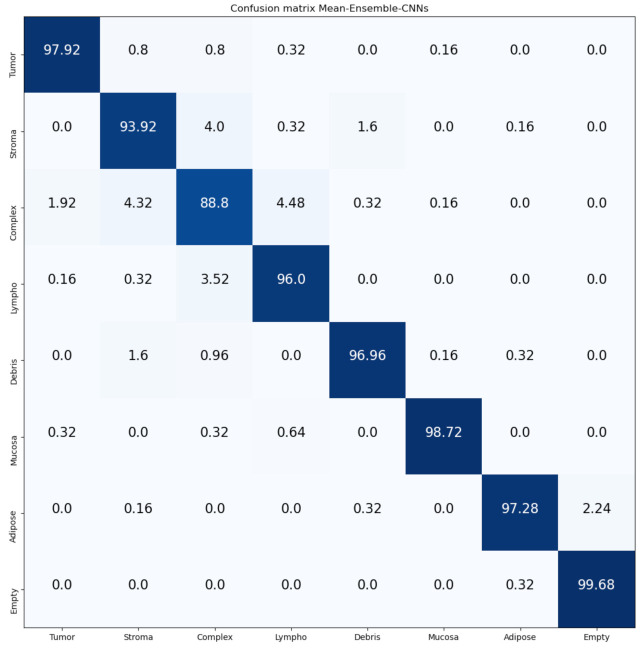
Confusion Matrix of our proposed approach: Mean-Ensemble-CNNs on Kather-CRC-2016 database. The vertical axis is for the true classes and the horizontal axis is for the predicted classes.

**Figure 15:**
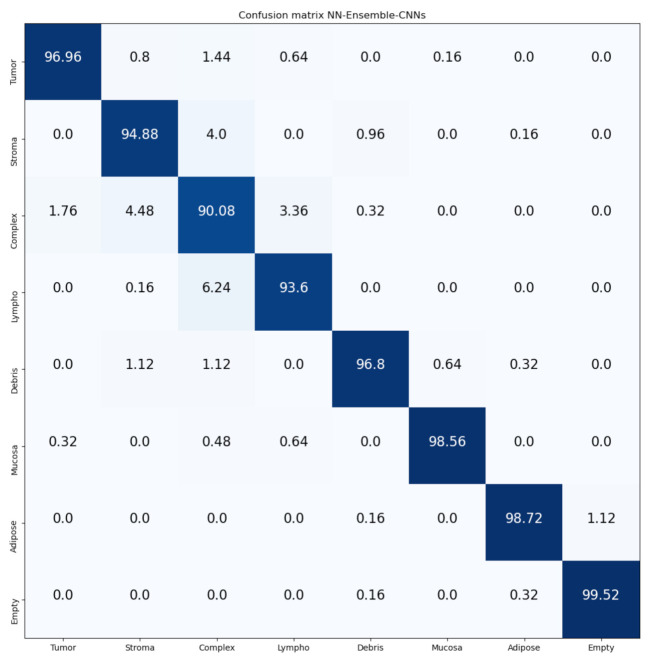
Confusion Matrix of our proposed approach: NN-Ensemble-CNNs on Kather-CRC-2016 database. The vertical axis is for the true classes and the horizontal axis is for the predicted classes.

Figure 14 and Figure 15 show the confusion matrices of our proposed Mean-Ensemble-CNN and NN-Ensemble-CNN approach, respectively. From these confusion matrices, we notice that both Ensemble approaches achieved close results in the recognition of each CRC tissue type on the Kather-CRC-2016 database.

#### 4.2.2. CRC-TP Database

To train the CNN architectures using the training data of CRC-TP database, we used batch size of 128. Similarly, we used batch size of 128 to train the four NN layers of our NN-Ensemble-CNN approach. In CRC-TP database experiments, we selected bigger batch sizes than the experiments of the Kather-CRC-2016 database because the CRC-TP database contains a larger number of samples for each class. Table 5 summarizes the obtained results using the CNN architectures and the proposed ensemble approaches. By comparing these results with the ones from Table 3, we notice that the CNN architectures exceed the hand-crafted feature-based methods in the classification of CRC tissue types. On the other hand, the performance of the two proposed ensemble approaches outperformed the performance of the four CNN architectures.

**Figure 16:**
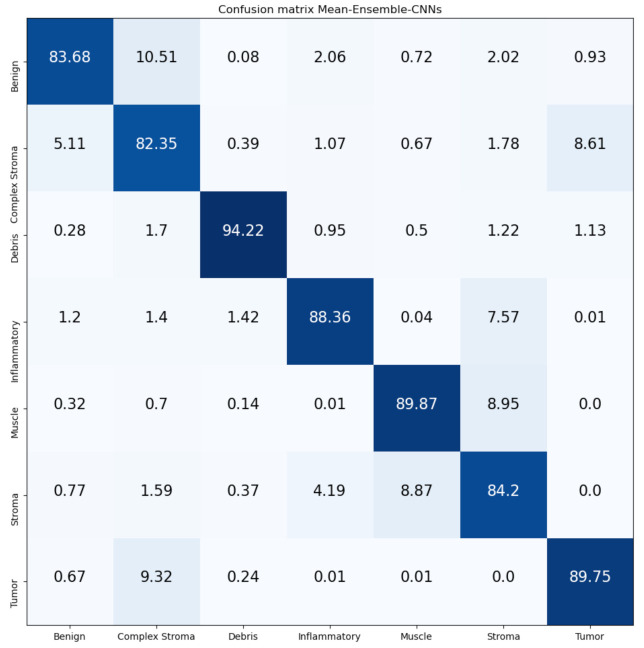
Confusion Matrix of Mean-Ensemble-CNNs on CRC-TP database. The vertical axis is for the true classes and the horizontal axis is for the predicted classes.

**Figure 17:**
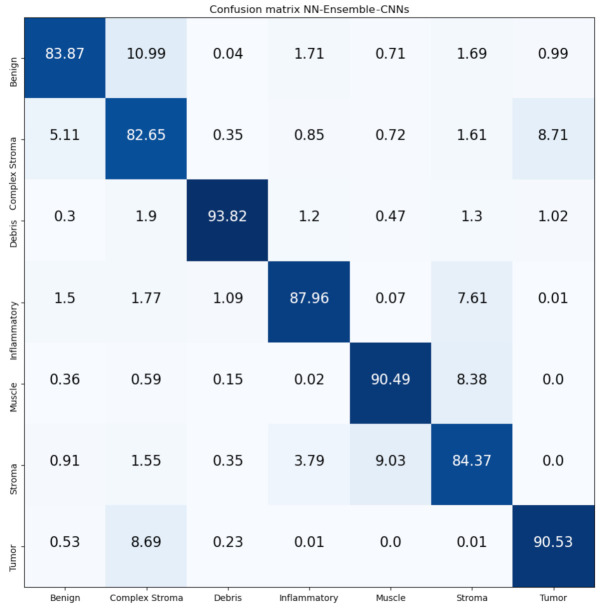
Confusion Matrix of NN-Ensemble-CNNs on CRC-TP database. The vertical axis is for the true classes, and the horizontal axis is for the predicted classes.

Figure 16 and Figure 17 show the confusion matrices of our proposed Mean-Ensemble-CNN and NN-Ensemble-CNN approaches. The comparison between the performance of the two proposed ensemble approaches (from Table 5 and Figure 16 and Figure 17) show that the NN-Ensemble-CNN approach performs slightly better on the recognition of CRC tissue types.

Since our approaches are an ensemble of trained CNN architectures, it is interesting to compare the computational cost of our proposed approaches with these CNN architectures. Table 6 contains the required time to test single CRC tissue type image using the trained CNN architectures and our approaches on Kather-CRC-2016 and CRC-TP databases. From Table 6, we notice that our approaches’ testing time is equal to the sum of single models’ testing times. Moreover, we notice that the required time is very trivial for all the evaluated methods in both databases. Therefore, our approaches are suitable for real-world digital pathology application.

## 5. Discussion

In this section, we will compare our results with state-of-the-art methods. Table 7 contains the comparison between our proposed approaches and the state-of-the-art methods. In [12], J. Kather et al. tested different texture descriptors with an SVM classifier. In [41], Ł. Rączkowski et al. proposed the Bayesian Convolutional Neural Network approach. In [19], L. Nanni et al. proposed an ensemble (FUS_ND+DeepOutput) approach based on combining deep and texture features. The comparison in Table 7 shows that our proposed ensemble approaches outperform the state-of-the-art methods.

Table 8 contains the comparison between our proposed approaches and the state-of-the-art methods on the CRT-TP database. In [8], S. Javed et al. used supervized and semi-supervized learning methods. In this comparison, we consider the results of the supervized approaches which are similar to our approaches. In Table 8, we compare our approaches with texture and deep learning methods that were tested on [8]. The comparison shows that our approaches (Mean-Ensemble-CNNs and NN-Ensemble-CNNs) outperform the state-of-the-art methods. The comparison with the hand-crafted feature-based methods, deep learning architectures and the state-of-the-art methods proves the efficiency of our proposed ensemble approaches (Mean-Ensemble-CNNs and NN-Ensemble-CNNs).

From the results on the Kather-CRC-2016 database, we notice that our proposed approaches (Mean-Ensemble-CNNs and NN-Ensemble-CNNs approach) achieved similar results (Table 7). Meanwhile, in the CRC-TP database, we notice that the NN-Ensemble-CNNs performance is better than Mean-Ensemble-CNNs (Table 8). On the other hand, we noticed that the performance of different methods on Kather-CRC-2016 is better than the performance on the CRC-TP database. This is probably because the CRC-TP database contains more challenging classes than Kather-CRC-2016. In addition, CRC-TP is not a balanced database that can influence the overall performance. Another possible reason can be the splitting and labeling of the tissue types, which were performed by different expert pathologists for each database. Despite our approach outperforming the state-of-the-art methods in both databases, the results in the CRC-TP database need more improvements for real-world applications. One possible solution is to use more data augmentation techniques to increase the training data.

## 6. Conclusions

In this paper, we proposed two Ensemble deep learning approaches to recognize the CRC tissue types. Our proposed approaches are denoted by Mean-Ensemble-CNNs and NN-Ensemble-CNNs, which are based on combining four trained CNN architectures. The trained CNN architectures are ResNet-101, ResNeXt-50, Inception-v3 and DenseNet-161. In our Mean-Ensemble-CNN approach, we combined the CNN architectures by averaging their predicted probabilities. In our NN-Ensemble-CNN approach, we combined the deep features from the last fully connected layer of each trained CNN architecture, then feed them into four layers NN. In addition to evaluating the four CNN architectures and our proposed approaches, we evaluated two texture descriptors and two classifiers. In more detail, we evaluated LPQ features, BSIF features and their combination by using two classifiers which are: SVM and NN.

The experimental results showed that deep learning methods (single architecture) surpass the hand-crafted feature-based methods. On the other hand, our proposed approaches outperform both the hand-crafted feature-based methods and the CNN architectures. In addition, our ensemble approaches outperform the state-of-the-art methods in both databases. As for future work, we are planning to use more data augmentation techniques to augment the training data. Moreover, including other powerful CNN architectures to our ensemble approaches will help to improve the performance.

## Figures and Tables

**Table 1:** CRC-TP database composition.

Class	Number of Images
Tumor	50,000
Complex Stroma	50,000
Stroma	50,000
Smooth Muscle	50,000
Benign	30,000
Inflammatory	30,000
Debris	20,000

**Table 2:** The accuracy results of LBQ and BSIF descriptors and their combination using SVM and NN classifiers on Kather-CRC-2016 database.

Model	Fold 1 (%)	Fold 2 (%)	Fold 3 (%)	Fold 4 (%)	Fold 5 (%)	Mean (%)
LPQ, SVM	69.60	67.60	67.70	68.30	67.40	68.12
BSIF, SVM	68.10	66.20	70.80	67.70	67.70	68.10
LPQ+BSIF, SVM	72.80	73.00	**76.30**	**74.10**	74.30	74.10
LPQ, NN	67.30	68.90	68.50	70.70	69.70	69.02
BSIF, NN	71.80	69.50	72.50	70.70	70.70	71.04
LPQ+BSIF, NN	**75.50**	**73.90**	74.20	73.10	**74.40**	**74.22**

**Table 3:** The results of LBQ and BSIF descriptors and their combination using SVM and NN classifiers on CRC-TP database.

Model	Accuracy (%)	$\hat{F1}$-Score (%)
LPQ, SVM	58.14	58.16
BSIF, SVM	56.45	56.40
LPQ+BSIF, SVM	63.40	63.25
LPQ, NN	61.40	61.34
BSIF, NN	61.16	61.17
LPQ + BSIF, NN	**65.56**	**65.43**

**Table 4:** Experimental results using ResNet-101, ResNeXt-50, Inception-v3, DenseNet-161, Mean-Ensemble-CNNs and NN-Ensemble-CNN on Kather-CRC-2016 database using the accuracy measurement.

Architecture	Fold 1 (%)	Fold 2 (%)	Fold 3 (%)	Fold 4 (%)	Fold 5 (%)	Mean (%)
ResNet-101	95.80	94.30	96.10	**97.50**	**95.90**	95.92
ResNeXt-50	96.20	94.00	95.80	97.10	95.60	95.74
Inception-v3	94.80	92.00	93.80	95.80	93.50	93.98
DenseNet-161	95.90	94.10	95.90	96.90	95.20	95.60
Mean-Ensemble-CNNs	96.20	95.00	**96.30**	**97.50**	95.80	**96.16**
NN-Ensemble-CNNs	**96.40**	**95.60**	96.00	97.10	95.60	96.14

**Table 5:** Experimental results using ResNet-101, ResNeXt-50, Inception-v3, DenseNet-161, Mean-Ensemble-CNNs and NN-Ensemble-CNN on CRC-TP database.

Architecture	Accuracy (%)	$\hat{F1}$-Score (%)
ResNet-101	85.98	85.99
ResNeXt-50	85.53	85.55
Inception-v3	85.50	85.46
DenseNet-161	86.28	86.30
Mean-Ensemble-CNNs	86.97	86.99
NN-Ensemble-CNNs	**87.26**	**87.27**

**Table 6:** Testing time for the evaluated CNN architectures (ResNet-101, ResNeXt-50, Inception-v3 and DenseNet-16) and our proposed approaches (Mean-Ensemble-CNNs and NN-Ensemble-CNNs) for each database.

Model	Databases	
	Kather-CRC-2016 Database (s)	CRC-TP Database (s)
ResNet-101	0.008598	0.037415
ResNeXt-50	0.009178	0.030339
Inception-v3	0.008513	0.020904
DenseNet-161	0.015966	0.038318
Mean-Ensemble-CNNs	0.042255	0.126976
NN-Ensemble-CNNs	0.042520	0.127373

**Table 7:** Comparison between our approaches and the state-of-the-art methods on Kather-CRC-2016 database.

Methods	N of Folds	Accuracy (%)
Gabor+rbf-SVM [12]	10	62.60
Perceptual+rbf-SVM [12]	10	63.00
GLCM+rbf-SVM [12]	10	71.90
Histogram higher+rbf-SVM [12]	10	72.40
LBP+rbf-SVM [12]	10	76.20
Histogram Lower+rbf-SVM [12]	10	80.80
ARA-CNN [41]	5	92.24
FUS_ND+DeepOutput [19]	5	93.24
Mean-Ensemble-CNNs (Our)	5	**96.16**
NN-Ensemble-CNNs (Our)	5	96.14

**Table 8:** Comparison between our approaches and the state-of-the-art methods on CRC-TP database. Where: Tu: Tumor, St: Stroma, CS: Complex Stroma, Be: Benign, De: Debris, In: Inflammatory and SM: Smooth Muscle. ${}^{*}$ are the comparison methods from [8].

Methods	Tu (%)	St (%)	CS (%)	Be (%)	De (%)	In (%)	SM (%)	$\hat{F1}$-Score (%)
Subspace Clustering ${}^{*}$	48	62	45	46	64	65	63	55
SCD ${}^{*}$	60	61	55	69	81	79	69	65
DL-KLdiv ${}^{*}$	62	65	60	79	73	76	70	68
SRC ${}^{*}$	73	75	65	60	85	66	64	69
SHIRC ${}^{*}$	78	75	61	65	68	78	69	71
KM-CD ${}^{*}$	72	79	62	73	80	78	79	73
DFOD ${}^{*}$	84	81	73	71	78	74	74	77
SDLs ${}^{*}$	86	83	70	72	81	80	70	77
SPM ${}^{*}$	82	80	70	85	83	84	74	79
B5F-SVM ${}^{*}$	86	77	73	75	91	92	78	80
MobileNet ${}^{*}$	79	79	68	81	76	82	76	77
SVM-CNN ${}^{*}$	80	78	80	73	84	86	79	80
ResNet-50 ${}^{*}$	81	81	78	81	88	87	85	82
Mean-Ensemble-CNNs (Our)	89.75	84.20	82.35	83.68	**94.22**	**88.36**	89.87	86.99
NN-Ensemble-CNNs (Our)	**90.53**	**84.37**	**82.65**	**83.87**	93.82	87.96	**90.49**	**87.27**

## Data Availability

The used datasets were obtained from publically open source datasets: 1- Kather-CRC-2016 Database https://zenodo.org/record/53169#.YEEFpY5KjyS. 2- CRC-TP Database https://warwick.ac.uk/fac/sci/dcs/research/tia/data/crc-tp.

## Abbreviations

The following abbreviations are used in this manuscript:

CNN	Convolutional Neural Network
RT-PCR	Reverse Transcription Polymerase Chain Reaction
SVM	Support Vector Machine
NN	Neural Network
LPQ	Local Phase Quantization
BSIF	Binarized Statistical Image Features
GLCM	Gray-Level Co-Occurrence Matrix
LBP	Local Binary Pattern

## References

[1] Farahani N., Parwani A.V., Pantanowitz L. (2015). Whole slide imaging in pathology: Advantages, limitations, and emerging perspectives. Pathol. Lab. Med. Int..

[2] Pantanowitz L., Sharma A., Carter A.B., Kurc T., Sussman A., Saltz J. (2018). Twenty years of digital pathology: An overview of the road travelled, what is on the horizon, and the emergence of vendor-neutral archives. J. Pathol. Inform..

[3] Egeblad M., Nakasone E.S., Werb Z. (2010). Tumors as organs: Complex tissues that interface with the entire organism. Dev. Cell.

[4] Huijbers A., Tollenaar R., Pelt G.W., Zeestraten E.C.M., Dutton S., McConkey C.C., Domingo E., Smit V., Midgley R., Warren B.F. (2013). The proportion of tumor-stroma as a strong prognosticator for stage II and III colon cancer patients: Validation in the VICTOR trial. Ann. Oncol..

[5] Marusyk A., Almendro V., Polyak K. (2012). Intra-tumour heterogeneity: A looking glass for cancer?. Nat. Rev. Cancer.

[6] Bray F., Ferlay J., Soerjomataram I., Siegel R.L., Torre L.A., Jemal A. (2018). Global cancer statistics 2018: GLOBOCAN estimates of incidence and mortality worldwide for 36 cancers in 185 countries. CA Cancer J. Clin..

[7] Sirinukunwattana K., Snead D., Epstein D., Aftab Z., Mujeeb I., Tsang Y.W., Cree I., Rajpoot N. (2018). Novel digital signatures of tissue phenotypes for predicting distant metastasis in colorectal cancer. Sci. Rep..

[8] Javed S., Mahmood A., Fraz M.M., Koohbanani N.A., Benes K., Tsang Y.W., Hewitt K., Epstein D., Snead D., Rajpoot N. (2020). Cellular community detection for tissue phenotyping in colorectal cancer histology images. Med. Image Anal..

[9] Nearchou I.P., Soutar D.A., Ueno H., Harrison D.J., Arandjelovic O., Caie P.D. (2021). A comparison of methods for studying the tumor microenvironment’s spatial heterogeneity in digital pathology specimens. J. Pathol. Inform..

[10] Bianconi F., Álvarez Larrán A., Fernández A. (2015). Discrimination between tumour epithelium and stroma via perception-based features. Neurocomputing.

[11] Linder N., Konsti J., Turkki R., Rahtu E., Lundin M., Nordling S., Haglund C., Ahonen T., Pietikäinen M., Lundin J. (2012). Identification of tumor epithelium and stroma in tissue microarrays using texture analysis. Diagn. Pathol..

[12] Kather J.N., Weis C.A., Bianconi F., Melchers S.M., Schad L.R., Gaiser T., Marx A., Zöllner F.G. (2016). Multi-class texture analysis in colorectal cancer histology. Sci. Rep..

[13] Javed S., Mahmood A., Werghi N., Benes K., Rajpoot N. (2020). Multiplex Cellular Communities in Multi-Gigapixel Colorectal Cancer Histology Images for Tissue Phenotyping. IEEE Trans. Image Process..

[14] CRCHistoPhenotypes. https://warwick.ac.uk/fac/cross_fac/tia/data/crchistolabelednucleihe.

[15] Kather J.N., Krisam J., Charoentong P., Luedde T., Herpel E., Weis C.A., Gaiser T., Marx A., Valous N.A., Ferber D. (2019). Predicting survival from colorectal cancer histology slides using deep learning: A retrospective multicenter study. PLoS Med..

[16] Kothari S., Phan J.H., Young A.N., Wang M.D. (2013). Histological image classification using biologically interpretable shape-based features. BMC Med. Imaging.

[17] Bejnordi B.E., Mullooly M., Pfeiffer R.M., Fan S., Vacek P.M., Weaver D.L., Herschorn S., Brinton L.A., van Ginneken B., Karssemeijer N. (2018). Using deep convolutional neural networks to identify and classify tumor-associated stroma in diagnostic breast biopsies. Mod. Pathol..

[18] Du Y., Zhang R., Zargari A., Thai T.C., Gunderson C.C., Moxley K.M., Liu H., Zheng B., Qiu Y. (2018). Classification of tumor epithelium and stroma by exploiting image features learned by deep convolutional neural networks. Ann. Biomed. Eng..

[19] Nanni L., Brahnam S., Ghidoni S., Lumini A. (2018). Bioimage classification with handcrafted and learned features. IEEE/ACM Trans. Comput. Biol. Bioinform..

[20] Bougourzi F., Dornaika F., Mokrani K., Taleb-Ahmed A., Ruichek Y. (2020). Fusion Transformed Deep and Shallow features (FTDS) for Image-Based Facial Expression Recognition. Expert Syst. Appl..

[21] Wang S., Yang D.M., Rong R., Zhan X., Fujimoto J., Liu H., Minna J., Wistuba I.I., Xie Y., Xiao G. (2019). Artificial intelligence in lung cancer pathology image analysis. Cancers.

[22] Ouahabi A., Taleb-Ahmed A. (2021). Deep learning for real-time semantic segmentation: Application in ultrasound imaging. Pattern Recognit. Lett..

[23] Cascianelli S., Bello-Cerezo R., Bianconi F., Fravolini M.L., Belal M., Palumbo B., Kather J.N. Dimensionality reduction strategies for cnn-based classification of histopathological images. Proceedings of the International Conference on Intelligent Interactive Multimedia Systems and Services.

[24] Simonyan K., Zisserman A. (2014). Very deep convolutional networks for large-scale image recognition. arXiv.

[25] Krizhevsky A., Sutskever I., Hinton G.E. (2017). Imagenet classification with deep convolutional neural networks. Commun. ACM.

[26] Iandola F.N., Han S., Moskewicz M.W., Ashraf K., Dally W.J., Keutzer K. (2016). SqueezeNet: AlexNet-level accuracy with 50x fewer parameters and <0.5 MB model size. arXiv.

[27] Szegedy C., Liu W., Jia Y., Sermanet P., Reed S., Anguelov D., Erhan D., Vanhoucke V., Rabinovich A. Going deeper with convolutions. Proceedings of the IEEE Conference on Computer Vision and Pattern Recognition.

[28] He K., Zhang X., Ren S., Sun J. Deep residual learning for image recognition. Proceedings of the IEEE Conference on Computer Vision and Pattern Recognition.

[29] Ojansivu V., Heikkilä J., Elmoataz A., Lezoray O., Nouboud F., Mammass D. (2008). Blur Insensitive Texture Classification Using Local Phase Quantization. Image and Signal Processing.

[30] Bougourzi F., Mokrani K., Ruichek Y., Dornaika F., Ouafi A., Taleb-Ahmed A. (2019). Fusion of transformed shallow features for facial expression recognition. IET Image Process..

[31] Kannala J., Rahtu E. BSIF: Binarized statistical image features. Proceedings of the 21st International Conference on Pattern Recognition (ICPR2012).

[32] Cortes C., Vapnik V. (1995). Support-vector networks. Mach. Learn..

[33] Xie S., Girshick R., Dollár P., Tu Z., He K. Aggregated residual transformations for deep neural networks. Proceedings of the IEEE Conference on Computer Vision and Pattern Recognition.

[34] Szegedy C., Vanhoucke V., Ioffe S., Shlens J., Wojna Z. (2015). Rethinking the Inception Architecture for Computer Vision. arXiv.

[35] Huang G., Liu Z., Van Der Maaten L., Weinberger K.Q. Densely connected convolutional networks. Proceedings of the IEEE Conference on Computer Vision and Pattern Recognition.

[36] Lin T.Y., Goyal P., Girshick R., He K., Dollár P. Focal loss for dense object detection. Proceedings of the IEEE International Conference on Computer Vision.

[37] Liu W., Chen L., Chen Y. (2018). Age Classification Using Convolutional Neural Networks with the Multi-class Focal Loss. IOP Conf. Ser. Mater. Sci. Eng..

[38] Bendjoudi I., Vanderhaegen F., Hamad D., Dornaika F. (2020). Multi-label, multi-task CNN approach for context-based emotion recognition. Inf. Fusion.

[39] Paszke A., Gross S., Massa F., Lerer A., Bradbury J., Chanan G., Killeen T., Lin Z., Gimelshein N., Antiga L. Pytorch: An imperative style, high-performance deep learning library. Proceedings of the Advances in Neural Information Processing Systems.

[40] Kingma D.P., Ba J. (2014). Adam: A method for stochastic optimization. arXiv.

[41] Rączkowski L., Możejko M., Zambonelli J., Szczurek E. (2019). ARA: Accurate, reliable and active histopathological image classification framework with Bayesian deep learning. Sci. Rep..

